# Irinotecan induces steroid and xenobiotic receptor (SXR) signaling to detoxification pathway in colon cancer cells

**DOI:** 10.1186/1476-4598-10-80

**Published:** 2011-07-06

**Authors:** Agnes Basseville, Laurence Preisser, Sophie de Carné Trécesson, Michèle Boisdron-Celle, Erick Gamelin, Olivier Coqueret, Alain Morel

**Affiliations:** 1 Cancer Center Paul Papin, INSERM U892 / University of Angers, 2 rue Moll, Angers, F-49033, France; 2 Medical Oncology Branch, Center for Cancer Research, National Cancer Institute, National Institues of Health, 9000 Rockville Pike, Bethesda, MD 20892, USA

## Abstract

**Background:**

Resistance to chemotherapy remains one of the principle obstacles to the treatment of colon cancer. In order to identify the molecular mechanism of this resistance, we investigated the role of the steroid and xenobiotic receptor (SXR) in the induction of drug resistance. Indeed, this nuclear receptor plays an important role in response to xenobiotics through the upregulation of detoxification genes. Following drug treatments, SXR is activated and interacts with the retinoid X receptor (RXR) to induce expression of some genes involved in drug metabolism such as phase I enzyme (like CYP), phase II enzymes (like UGT) and transporters (e.g. MDR1).

**Results:**

In this study, we have shown that endogenous SXR is activated in response to SN-38, the active metabolite of the anticancer drug irinotecan, in human colon cancer cell lines. We have found that endogenous SXR translocates into the nucleus and associates with RXR upon SN-38 treatment. Using ChIP, we have demonstrated that endogenous SXR, following its activation, binds to the native promoter of the CYP3A4 gene to induce its expression. RNA interference experiments confirmed SXR involvement in CYP3A4 overexpression and permitted us to identify CYP3A5 and MRP2 transporter as SXR target genes. As a consequence, cells overexpressing SXR were found to be less sensitive to irinotecan treatment.

**Conclusions:**

Altogether, these results suggest that the SXR pathway is involved in colon cancer irinotecan resistance in colon cancer cell line via the upregulation of select detoxification genes.

## Background

One of the challenges in cancer treatment is to understand why some tumors fail to respond to chemotherapy. Delineating in advance the subsets of tumors presenting treatment failure and identifying which pathways are involved in drug resistance would thus represent a significant advance. Several factors contribute to the development of drug resistance. Inadequate drug access to the tumor, drug metabolism and excretion, activation of DNA repair mechanisms, and inactivation of cell death pathways have all been proposed as potential mechanisms used by tumor cells to escape treatment [1,2].

Drug metabolism reactions are divided into three phases: functionalization (phase I enzyme), conjugation (phase II enzymes), and transport (phase III proteins), but it is essentially carried out by cytochrome p450 3A4 (CYP3A4), which metabolizes more than 50% of all administered drug [3]. CYP3A4 is the predominant isoform of monooxygenases present in the liver but there is also evidence that metabolism occurs within the tumors that express this isoform, and thereby reduces the efficacy of chemotherapeutic agents [4,5]. It has been demonstrated that the transcriptional regulation of the CYP3A4 gene was mediated by the steroid and xenobiotic receptor SXR, also known as the nuclear receptor PXR (pregnane X receptor) [6-9]. SXR is a nuclear receptor mainly expressed in intestine and liver [9]. Following its activation by xenobiotics such as rifampicin, SXR interacts with the retinoid X receptor (RXR) to induce the transcriptional activation of several genes involved in drug metabolism [6,9]. In humans, SXR has been reported to bind the promoter and upregulate the expression of several CYPs (CYP3A, CYP2B and CYP2C) [7,9-15], the UDP-glucuronosyltransferase 1A1 (UGT1A1) [16], as well as the xenobiotic transporters multidrug resistance 1 (MDR1) and organic anion transporter 2 [17,18]. For these reasons, SXR is believed to play an important role in the defense against drugs by upregulating the expression of detoxification genes.

Irinotecan (or CPT-11), a camptothecin derivative, is one of the major drugs used in the treatment of colorectal cancers [19]. Irinotecan is a prodrug that forms the pharmacologically active compound 7-ethyl-10-hydroxycampto-thecin (or SN-38) via carboxylesterases 1 and 2 (CE1, CE2), but mostly by CE2 [20]. This agent then interacts with DNA topoisomerase I to induce the formation of cleavage complexes that prevent DNA replication. The collision of trapped topoisomerases with DNA replication forks induces DNA double strand breaks that finally lead to cell cycle arrest and cell death [21]. Irinotecan undergoes extensive metabolism: in both the liver and the intestine, it is converted to inactive metabolites by CYP3A4 and CYP3A5 [5,22] while its derivative SN-38 is inactivated through glucuronidation via UGT1A1, UGT1A6, UGT1A7 or UGT1A9 [23]. Irinotecan and its metabolites are also subject to detoxification by different export pumps like MDR1, breast cancer resistance protein (BCRP) and multidrug resistance proteins 1 and 2 (MRP1, MRP2) [24-28]. Since CYP3A4 upregulation is an important mechanism of drug resistance, these observations suggest that the SXR transcription factor could play an important role in tumor escape to irinotecan treatment through the upregulation of CYP3A4 and drug detoxification.

In this study, we identified SN-38, the active metabolite of irinotecan, as a new activator of SXR and elucidated a molecular mechanism by which colon cancer cells might acquire resistance. Upon drug treatment of colon cancer cell lines, SXR is translocated into the nucleus and interacts with RXR. Then, the SXR/RXR heterodimer binds to the promoter of the CYP3A4 gene to induce its expression. As a consequence, cells overexpressing the SXR transcription factor appear to be significantly less sensitive to irinotecan, perhaps due to an enhanced expression of CYP3A4 leading to irinotecan inactivation.

Altogether, these results reveal a complex network of interactions indicating that the SXR pathway induces the expression of detoxification genes in response to the topoisomerase I inhibitor, thereby leading to enhance drug resistance.

## Results

### SXR is recruited to the nucleus after SN-38 treatment

**Figure 1:**
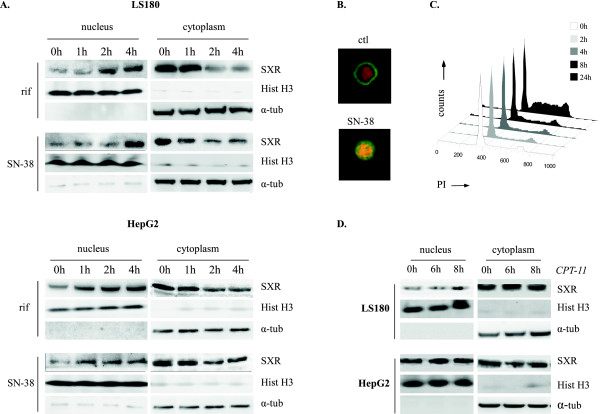
**SXR accumulates in the nucleus after SN-38 treatment**. A. LS180 and HepG2 cells were treated with 10 μM rifampicin (rif) or 10 ng/ml SN-38 for the indicated times, then nuclear and cytoplasmic extracts were prepared and subjected to WB with the indicated antibodies. B. After for 4 h exposure to 10 ng/ml SN-38, immunofluorescence experiment were performed in LS180 cells, showing SXR (green) and nucleus (red). Yellow colour indicates colocalization. C. Analysis of the cell cycle was performed by flow cytometry in LS180 cells after SN-38 exposure. D. LS180 and HepG2 cells were treated with 1 μM CPT-11 then nuclear and cytoplasmic extracts were prepared and subjected to WB as above.

**Figure 2:**
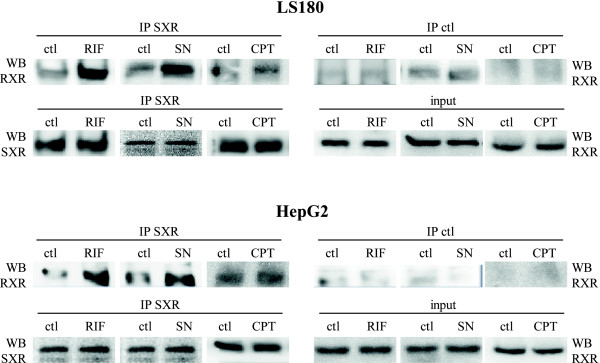
**SXR interacts with RXR in cells in a SN-38-dependent manner**. LS180 and HepG2 cells were treated with 10 μM rifampicin (RIF), 10 ng/ml SN-38 (SN) or 1 μg/ml CPT-11 (CPT) for 4 h. Cell extracts were immunoprecipitated (IP) with either IgG (IP control) or anti-SXR (IP SXR) in the presence of vehicle (ctl), rifampicin, SN-38 or CPT-11. Association of the endogenous RXR with the anti-SXR precipitate was detected by WB using anti-RXR antibody. Input indicates endogenous RXR present in 5% of total cell lysates used in each IP. To ensure equal amounts were precipitated, WB using anti-SXR was also performed.

In order to determine if SXR is activated by CPT-11, or its active derivative SN-38, we have studied cellular localization of the endogenous nuclear receptor SXR during CPT-11 and SN-38 treatment. Since it has been previously reported that rifampicin stimulates SXR-mediated transcription [6,9], this drug was used as a positive control in our cell lines. Following stimulation, cytoplasmic and nuclear extracts were recovered from the colon cancer cell line LS180, used as drug target cell model, and the hepatic cancer cell line HepG2, used as the major site for irinotecan metabolism. Western blot experiments were then performed with antibodies directed against SXR, the nucleus marker histone H3 and the cytoplasmic marker α-tubulin (Figure 1A and 1D). Before drug treatment, SXR was mainly cytoplasmic in LS180 cells and equally distributed between the cytosolic and nuclear compartments in HepG2 cells. We observed that rifampicin activated SXR by inducing its nuclear translocation after a 2 h stimulation in both cell lines. Interestingly, western blot experiments showed that SN-38 also induced the nuclear translocation of SXR, which was correlated with decreased cytosolic content (Figure 1A). Despite nuclear recruitment being observed in both cell lines after SN-38 and rifampicin treatment, it was stronger in the colon cell line. SXR nuclear recruitment was confirmed by confocal microscopy experiment in LS180 cells after 4 h exposure to SN-38 (Figure 1B). In order to determine if the SXR activation was an indirect consequence of cell cycle arrest, we performed cell cycle analysis by flow cytometry in LS180 treated with SN-38 (Figure 1C). Cell cycle arrest was not observed until 8 h drug exposure, indicating that it was not a cause of SXR activation. Moreover, SXR expression was not induced after 4 h drug exposure, as observed by WB in figure 2, meaning that the increase of SXR level in the nucleus was not due to its overexpression. No significant variation in SXR localisation was noticed after 8 h exposure to CPT-11 (Figure 1D).

Thus, we concluded that SN-38 but not CPT-11 induced SXR nuclear recruitment in colon and hepatic cell lines.

### SXR interacts with the retinoid X receptor after SN-38 treatment

SXR belongs to the same subfamily of nuclear receptors as thyroid hormone receptors (TRs) and retinoic acid receptors (RARs) which form heterodimers with RXR to mediate ligand-dependent transcription [29]. To determine if the nuclear translocation of SXR upon genotoxic treatment induced its association with RXR, LS180 and HepG2 cells were stimulated with 10 μM rifampicin, 10 ng/ml SN-38 and 1 μg/ml CPT-11 during 4 h. Coimmunoprecipitations were performed with anti-SXR antibody and the proteins present in the immunoprecipitates were revealed by immunoblotting with the RXR antibody (Figure 2). As expected, endogenous SXR and RXR were found to co-immunoprecipitate following 4 h of rifampicin stimulation. Interestingly, the same association was also observed after 4 h of SN-38 treatment. This interaction was dependent on the presence of the drugs since a very weak interaction was detected between the two proteins in non-stimulated cells. No interaction between SXR and RXR was observed after 4 h of CPT-11 exposure in both cell lines (Figure 2).

We infer from these results that SXR interacted with RXR upon SN-38 treatment in LS180 and HepG2 cells whereas no interaction has been reported after CPT-11 treatment.

### SXR interacts with the native cytochrome p450 3A4 promoter after SN-38 treatment

**Figure 3:**
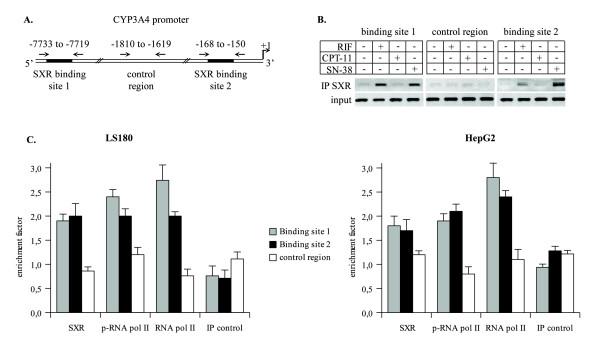
**SN-38 treatment induces binding of endogenous SXR to the native CYP3A4 promoter**. A. ChIP assays were performed and DNA was further analyzed either by classical PCR or by semi-quantitative PCR using a primer set specific for the promoter or control region as indicated by the arrows on the schematic diagram of the CYP3A4 promoter. B. LS180 were treated with 10 μM of rifampicin (rif), 1 μg/ml CPT-11 or 10 ng/ml SN-38 for 4 h and subjected to formaldehyde cross-linking. Soluble chromatin was prepared by sonication. Precleared chromatin solution was immunoprecipitated by antibodies anti-SXR or Ig control, and precipitated. PCR was performed with the precipitated DNA (IP SXR) or the DNA present in 10% of total cell lysates used for each IP (input). C. Cells were treated with SN-38 for 4 h and subjected to ChIP as described above with anti-SXR, anti-RNA phospho-ARN polymerase II and anti-RNA polymerase II. Enrichment factors were determined by qPCR using GAPDH as threshold indicator (internal control for each IP).

The formation of heterodimers between SXR and RXR is expected to mediate DNA binding to xenobiotic-response elements (XREs) present on SXR target genes such as CYP3A4. To verify this observation, we determined by chromatin immunoprecipitation (ChIP) experiments whether SN-38 treatment induced the binding of the transcription factor to one of its target genes, namely the CYP3A4 promoter. Two XREs were previously found at the -7773/-7719 and -169/-150 regions of the promoter and were named binding site 1 and 2 respectively (Figure 3A). Previous studies had shown that the two SXR binding sites worked in a synergistic way but could induce CYP3A4 transcription separately. Nevertheless, the binding site 2 was necessary to the maximal activation [7,9].

Chromatin was prepared using a formaldehyde cross-linking protocol and occupancy of the promoter was analyzed using specific pairs of primers spanning the two XREs. As controls, PCR analysis was also performed with a third set of primers spanning a promoter unrelated region (Figure 3A), and immunoprecipitations were conducted with irrelevant IgG to ensure the specificity of the reaction. As expected, antibodies directed against SXR precipitated DNA encompassing the two SXR responsive elements of the CYP3A4 promoter following rifampicin stimulation. Importantly, we also observed that SXR was recruited to both XREs upon SN-38 treatment but not upon CPT-11 treatment (Figure 3B). As a control, PCR analysis did not detect any occupancy of the control region located on the CYP3A4 promoter. Quantitative PCR analysis indicated that SXR bound with the same affinity to the two XREs (Figure 3C).

Increased binding of the transcription factor is expected to facilitate the recruitment of the RNA polymerase II to the CYP3A4 promoter. To test this hypothesis, ChIP experiments were also performed using antibodies directed against the RNA polymerase II and its active phosphorylated form. As expected, quantitative PCR analysis indicated that the total RNA polymerase II as well as the phosphorylated form were recruited to the both SXR binding sites on CYP3A4 promoter after SN-38 stimulation. Similar results were obtained by using primers spanning the transcription start site (data not shown). The binding of RNA polymerase was detected on both XREs, suggesting that the recruitment of SXR could induce cooperative DNA binding and the formation of a functional enhanceosome.

Altogether, these results indicate that the SXR transcription factor bound to the native promoter of the CYP3A4 gene in association with the activated RNA polymerase II upon SN-38 treatment, but not upon CPT-11 treatment.

### SXR is involved in the SN-38-mediated upregulation of select irinotecan detoxification genes

To extend our results, we investigated the role of SXR in regulation of the genes involved in irinotecan metabolism. We first identified by RT-qPCR which detoxification genes were upregulated after CPT-11 and SN-38 treatment and then, after SXR expression inhibition, we determined which of the upregulated genes were induced by activation of the nuclear receptor.

**Figure 4:**
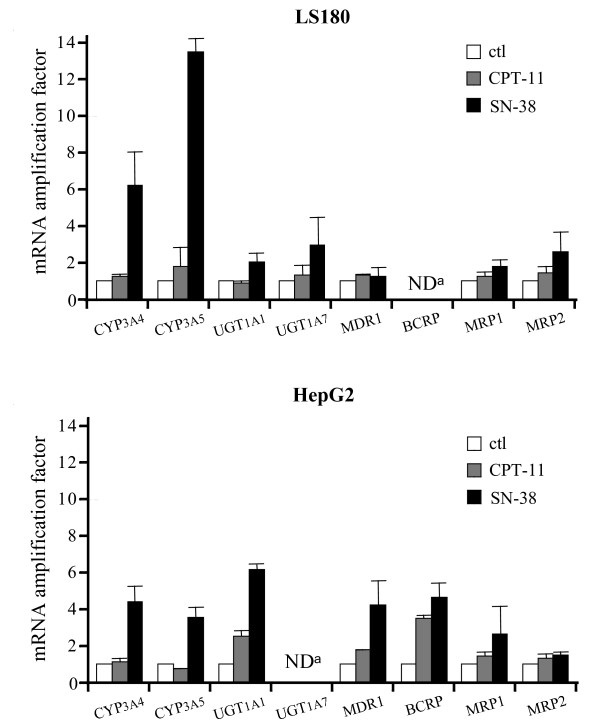
**Induction of irinotecan metabolism genes after CPT-11 and SN-38 treatment**. LS180 and HepG2 cells were treated with 1 μg/ml CPT-11 or 10 ng/ml SN-38 for 24 h and quantitative RT-PCR were performed on irinotecan metabolism genes. Fold inductions were calculated relative to the mean expression of GAPDH and experiments were performed in triplicate. (^a ^ND for not detected).

To this end, LS180 and HepG2 cells were treated during 24 h with CPT-11 and SN-38. Quantitative PCR experiments were then performed to detect the mRNA expression of the irinotecan metabolism phase I genes (CYP3A4, CYP3A5), the main irinotecan metabolism phase II genes (UGT1A1, UGT1A7) and the irinotecan transporters genes (MDR1, MRP1, MRP2 and BCRP). As presented in figure 4, in LS180 cells, SN-38 induces a strong increase in CYP3A4 and CYP3A5 transcription (respectively 6 and 13 times) and a weaker effect was observed for UGT1A1, UGT1A7 and MRP2 (~2-3 times) while CPT-11 treatment has no effect. In HepG2 cells, upon SN-38 treatment, CYP3A4, CYP3A5, MDR1 and BCRP mRNA were increased 4 times, UGT1A1 6 times and MRP1 two fold, whereas only UGT1A1 and BCRP genes were upregulated (~2-3 times) after CPT-11 treatment.

**Figure 5:**
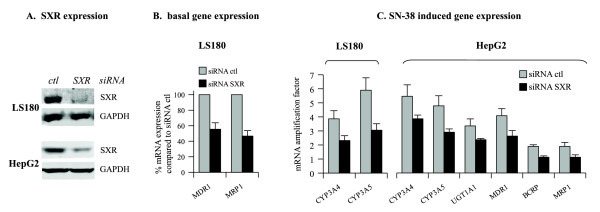
**SXR involvement in induction of irinotecan metabolism genes**. A. Detection of SXR by western blot in LS180 and HepG2 cells after 48 h siRNA transfection. B. After 48 h siRNA transfection, cells were cultivated for 24 h in new medium and mRNA levels of the irinotecan metabolism genes were determined. Data represent percentage of mRNA levels after siRNA-SXR transfection compared to siRNA-control transfection. C. After 48 h siRNA transfection, cells were stimulated with 10 ng/ml SN-38 for 24 h and mRNA levels were determined. Data represent fold induction of mRNA levels compared to untreated cells.

We then investigated the role of SXR on the CPT-11 and SN-38-mediated upregulated genes. To this end, LS180 and HepG2 cells were transiently transfected with control siRNA or siRNA targeting SXR prior to stimulation. As shown in figure 5A, siRNA transfection resulted in a significant reduction in SXR levels in LS180 cells (79% protein inhibition, measured by densitometry and relative to GAPDH expression) and a weaker reduction in HepG2 (55% inhibition). Interestingly, in LS180 cells, SXR knockdown led to a 50% decrease of MDR1 and MRP1 basal expression (Figure 5B). No significant effect of SXR downregulation was noticed on the basal expression of all the genes tested in HepG2. Moreover, reduction of SXR levels by siRNA prevented the upregulation of CYP3A4 and CYP3A5 following SN-38 treatment in LS180 (Figure 5C). The other tested genes showed no appreciable expression change after SXR downregulation (data not shown). In HepG2 cells, we observed that SXR knockdown blunted the SN-38-induced expression of most of the genes involved in irinotecan metabolism (Figure 5C). In addition, SXR downregulation had no effect on CPT-11-induced gene expression (data not shown).

Altogether, these results indicate that the SXR transcription factor activated the expression of the CYP3A4 and CYP3A5 gene in response to SN-38 in HepG2 and LS180 cells. MDR1 and MRP1 expression also appeared to be regulated by SXR in both cell lines while UGT1A1 and BCRP gene were regulated in a cell dependent manner.

### SXR promotes CPT-11 resistance in some colon cancer cell lines

**Figure 6:**
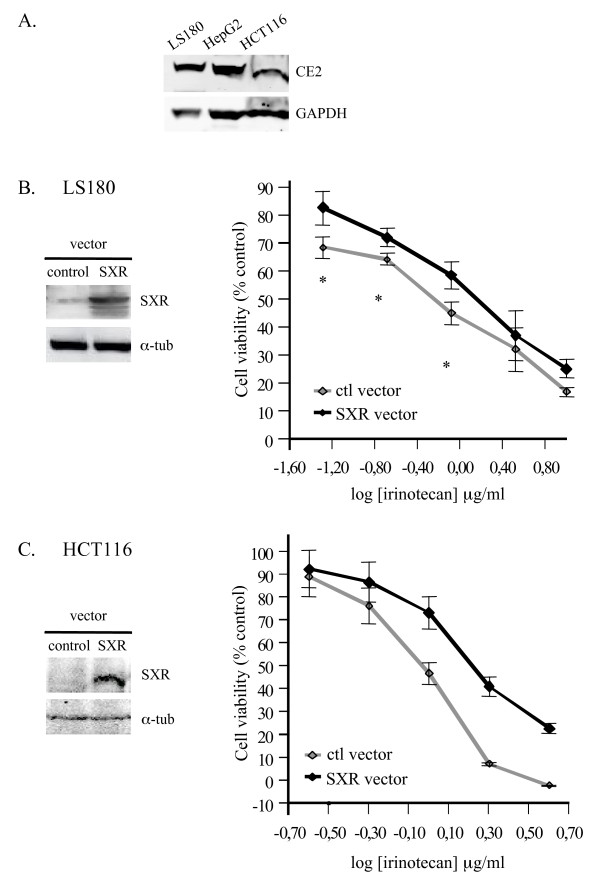
**Effect of SXR overexpression on LS180 and HCT116 survival after SN-38 treatment**. A. Detection of carboxylesterase 2 (CE2) by western blot in LS180, HepG2 and HCT116 cells. B. and C. LS180 cells (B) and HCT116 cells (C) were transfected with control or SXR vector for 24 h, then treated for 72 h with different CPT-11 concentrations. The SXR overexpression was confirmed by western blot after 24 h transfection. Cell viability following irinotecan exposure was determined by SRB assay by comparison with untreated cells. Each point represents the mean of 3 replicates and the experiment has been repeated 3 times. All results are expressed as the mean ± SE and obtained data were analysed for statistical differences by Student's t test. A p value of less than 0.05 was considered statistically significant (star indicates p < 0.05).

Since it is CPT-11 and not SN-38 which is converted to inactive metabolites through CYP3A4 and CYP3A5, the SXR-mediated upregulation of these genes should prevent the effect of CPT-11 on cell death. As a consequence, cells overexpressing SXR should be less sensitive to the topoisomerase inhibitor. To test this hypothesis, LS180 cells and HCT116 cells, another colon cancer cell line which not express SXR, were transiently transfected with SXR or control expression vectors for 24 h, then treated with increasing concentrations of irinotecan for 72 h (Figure 6). We first verified that the cells expressed the CE2 needed for CPT-11 conversion to the SXR activator SN-38 (Figure 6A). Then, the cytotoxicity assays confirmed that CPT-11 induced a dose-dependent decrease of cell survival in control conditions and that overexpression of SXR significantly reduced irinotecan induced cell death in LS180 cells (Figure 6B), as well as in HCT116 cells (Figure 6C).

Altogether, these results suggest that SXR overexpression limited the effect of CPT-11 on colon cancer cell death.

## Discussion

In addition to the regulation of tumor suppressor networks and DNA repair pathways, the ability of cancer cells to survive genotoxic treatments also relies on the efficacy of their detoxification pathways. In this study, we show for the first time that the nuclear receptor SXR is activated by SN-38, the active derivative of irinotecan, whereas CPT-11 itself is a very weak activator. Upon SN-38 treatment, endogenous SXR translocates into the nucleus, interacts with RXR and the so-formed heterodimer binds the CYP3A4 promoter, allowing the recruitment of the RNA polymerase and the expression of the CYP3A4 gene. These molecular mechanisms operate in colon cancer cells, a target of irinotecan treatment as well as in liver cancer cells, a major site of irinotecan metabolism.

**Figure 7:**
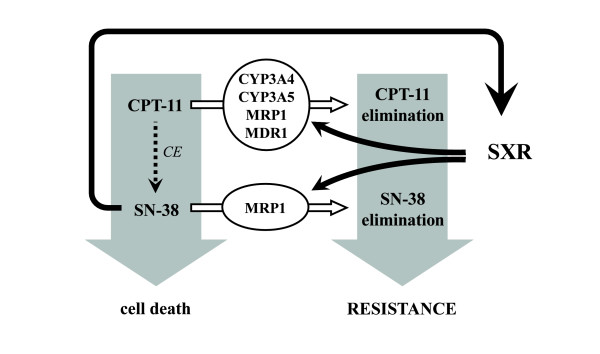
**Proposed model for the SXR-mediated response to irinotecan in colon cancer cells**. In LS180 cells, CPT-11 is metabolized to SN-38 by the carboxylesterases (CE). SN-38 induces the activation of SXR. Consequently, the transcription factor is recruited to the CYP3A4 and CYP3A5 promoter and upregulates the expression of these genes. SXR also constitutively activates MDR1 and MRP1 expression. As a feedback loop, CYP3A4, CYP3A5 and the transporters reduce the concentration of active irinotecan inside the cell, which favors cell proliferation. As a consequence, we propose that the combined detection of SXR together with a high expression of CYP3A4 should help to define in advance the subsets of tumors that will fail to respond to chemotherapy based on irinotecan treatment.

Moreover, we show that LS180 colon cancer cells that overexpress SXR are less sensitive to CPT-11 treatment compared to control cells, indicating that this transcription factor is involved in drug resistance, probably through CYP3A4, CYP3A5, MDR1 and MRP1 upregulation. In light of these observations, we propose a feedback model (Figure 7) in which the SXR-CYP3A pathway is induced in response to irinotecan treatment in colon cancer cells, SXR mediated expression of detoxification genes allowing for drug resistance and tumor escape to genotoxic treatments.

We observe some differences between the LS180 and HepG2 cellular models. Indeed, expression analyses of irinotecan detoxification genes (CYP3A, UGT1A and ABC transporters) show that most of these genes are induced upon SN-38 treatment, but their expression pattern varies depending on cell type. Furthermore, we find that HepG2 cells are more resistant to treatment: after 72 h incubation, IC50 values are 3.7-fold higher for CPT-11 and 5-fold higher for SN-38 in HepG2 than in LS180 cells (data not shown). This discrepancy can be explained by the upregulation of a large number of detoxification genes in the hepatic model, leading to stronger drug inactivation.

Moreover, SXR knockdown blunted but not abrogated the increase of the select genes that were induced by SN-38 treatment. We can hypothesize that the gene transcription could be induced by other transcription factors in addition to SXR. Thereby, the absence of SXR could allow other constitutively active nuclear receptors to access SXR target gene promoters. For example, it has been demonstrated that CAR and SXR can be activated by some common ligands and induce the same genes (CYP3A and CYP2B) by using the same consensus sites [30]. Other studies have established that FXR and VDR could share some target gene binding sites with SXR (reviewed by Zhou *et al*. [31]). All these receptors are mainly expressed in liver and/or colon cells.

It is interesting to note that gene regulation by SXR depends not only on the cell line but also on the SXR activator drug. For example MDR1, which is known to be a SXR target gene, does not exhibit any expression change after SN-38 treatment in LS180 whereas it is strongly induced by SXR upon rifampicin stimulation in the same cells (data not shown). Masuyama *et al*. [32] explain this specificity of gene induction by involvement of transcription co-activator proteins. Depending on the ligand, SXR would interact with different co-activators, leading to the induction of specific genes. In addition, Zhou *et al*. [33] have demonstrated that different co-repressors could also be involved in tissue-specific gene induction by SXR.

Surprisingly, if SN-38 is a SXR activator, CPT-11 is not. Assuming that there is a direct binding of these compounds on SXR, specific activation could be due to differences in ligand affinities for the receptor. This is surprising considering the very close structure of these two compounds and the SXR ligand binding pocket ability to welcome a great number of chemically different molecules [34]. Such differences have already been described between paclitaxel and docetaxel [35]. The weak impact of CPT-11 on metabolism gene induction could be due to its requirement to be converted into its active form SN-38 by CE2, which is expressed in HepG2 and LS180 cells. Moreover, Xu *et al*. [36] have evaluated CE2 expression in different tumors and have shown that 66% of tumor tissues expressed CE2 and that conversion of CPT-11 to SN-38 was correlated with the enzyme expression.

Knowing that detoxification is not the only mechanism involved in resistance to irinotecan, it will be interesting to determine if other genes are involved in the SXR mediated resistance. We have showed that the target gene (TOPO1) expression was not modified after treatment (data not shown). Irinotecan resistance could also be related to the changes of expression of DNA repair genes, cell cycle genes, cell death and survival pathway genes. Recently, two teams studied the anti-apoptotic role of SXR in some cancer cell lines [37,38]. They both demonstrated that overexpressed SXR in human cell lines protected against induced apoptosis and promoted drug resistance in connection with the upregulation of some anti-apoptotic genes and downregulation of several pro-apoptotic genes. Nevertheless, other studies reported a pro-apoptotic role of human SXR in breast cancer cells [39] or in colon cancer cells [40] but, as seen in a previous paragraph, this difference of gene regulation can be explained respectively by cell-type specificity and ligand specificity of SXR induction.

We have observed that SXR is involved in irinotecan detoxification. Other studies implicating SXR in anticancer drug detoxification like paclitaxel, cisplatin, tamoxifen or etoposide are already available [35,41-44]. Its involvement in resistance to paclitaxel and vinblastine in prostate cancer is clearly observed by Chen *et al*. [45]. In addition, a recent study showed that SXR was overexpressed in colon cancer tissues and suggested that the nuclear receptor may play role in resistance to 5-fluorouracil and oxaliplatin, two drugs used in treatment of colorectal cancer [46]. Those data clearly corroborate our work by demonstrating the influence of SXR on antineoplastic agents.

In addition, the ability of SXR to be activated by diverse therapeutic compounds places it as a major component in drug-drug interaction. This nuclear receptor is induced by a wide range of drugs: the antibiotic rifampicin, the anticancer drugs cited above, the barbiturate phenobarbital, the corticoid dexamethasone, the HIV protease inhibitor ritonavir or the antidepressant hyperforine [9,47-49]. Consequently, during a co-administration, SXR activation, leading to the induction of its target genes, could cause an accelerated metabolism of one of the drugs by the CYP3A4. This could lead to a diminution of clinical efficacy and could be at the origin of strong resistances. For example, a drug-drug interaction has been observed in some patients with glioma who have received CPT-11 and dexamethasone [50,51]. The discovery of SN-38 as a new activator of SXR may help prevent some cases of drug-drug interaction unsuspected until now. Considering that one of the SXR target gene, CYP3A4, is alone responsible for the metabolism of more than 50% of the drugs currently administrated, the search of novel partners of SXR is clearly relevant.

## Conclusions

All together, these results uncover new functions for the SXR pathway in the understanding of resistance to irinotecan. Our results suggest that tumors expressing SXR might be more resistant to anticancer treatment. We therefore propose that the SXR pathway should be considered as a valuable tool to predict the subsets of tumors that will fail to respond to chemotherapy.

## Methods

### Cell lines and treatment

LS180, HepG2 and HCT116 cells were purchased from the ATCC (Manassas, VA, USA). Experiments were done in MEM supplemented with 10% fetal bovine serum (Invitrogen, Carlsbad, CA, USA). Rifampicin and irinotecan were purchased from Sigma-Aldrich (St. Louis, MO, USA) and SN-38 from Aventis (Bridgewater, NJ, USA).

### Nuclear and cytoplasmic and total extracts

The cell pellets were suspended in buffer A (10 mM Hepes pH 7.9, 10 mM KCl, 1.5 mM MgCl2), kept on ice for 15 min, then subjected to 3 freeze-thaw cycles. The nuclei were pelleted by centrifuging at 400 g for 5 min at 4°C while supernatant was conserved as cytoplasmic fraction. Nuclei were washed with 1 ml buffer A, centrifuged at 400 g for 5 min at 4°C, resuspended in buffer C (20 mM Hepes, 420 mM KCl, 1.5 mM MgCl2, 0.2 mM EDTA, 25% glycerol) and kept on ice for 30 min. The nuclear fraction was then submitted to 10 s pulse sonication, centrifuged at 14000 g and supernatant were collected.

For total extract, cells were lysed in buffer C and incubated for 30 min on ice. Lysates were submitted to 10 s sonication and centrifugated at 15000 g during 5 min. Supernatant was collected and protein concentrations were determined by the Bradford method with Bio-Rad's Protein Assay Reagent (Bio-Rad, Hercules, CA) using BSA standards (Pierce, Rockford, IL).

### Western Blot

Protein extracts were loaded on 10% SDS/PAGE gels then proteins were transferred to PVDF membranes. The membranes were blocked with 5% defatted milk for 1 h, immunoblotted overnight at 4°C with anti-SXR (Santacruz Biotechnology, Santa Cruz, CA, USA or Abcam, Cambridge, MA, USA), anti-histone H3 (Cell Signaling, Beverly, MA, USA), anti-α-tubulin (Sigma), or anti-GAPDH (American Research Products, Belmont, MA, USA), then incubated with the secondary HRP-conjugated antibody. Bands were visualized using ECL detection reagents (Amersham Biosciences, Pittsburgh, PA, USA) and quantified with the ChemiDoc™ XRS imager (Bio-Rad, Hemel Hempstead, UK).

### Immunofluorescence

LS180 cells were fixed with 2% paraformaldehyde in PBS for 30 min at room temperature then permeabilized for 30 min in 70% ethanol. After blocking with 1% BSA, indirect immunostaining was performed using an anti-SXR antibody (Santa Cruz Biotechnology) and a fluorescein isothiocyanate-conjugated secondary antibody (Santacruz Biotechnology). Nuclei were stained with propidium iodide. Cells were observed using an Olympus confocal microscope (Olympus, Rungis, France), with high magnification (x630).

### Cell cycle assay

LS180 cells were permeabilised and fixed with ethanol 70%, treated with RNase A for 30 min at 37°C then incubated 5 min with propidium iodide. Cell cycle analysis was performed on a FACSort flow cytometer (BD Biosciences, San Jose, CA, USA) and data were analysed with Flowjow software (TreeStar, Inc., Ashland, OR, USA).

### Coimmunoprecipitation Assays

HepG2 and LS180 cells were treated with 10 μM rifampicin, 1 μg/ml CPT-11 or 10 ng/ml SN-38 for 4 h. Harvested cells were suspended in lysis buffer (20 mM Hepes pH 8, 0.2 mM EDTA, 5% glycerol, 100 mM NaCl, 0.1% nonidet P-40, 0.25% sodium deoxycholate, 1 mM NaF, 1 mM sodium orthovanadate, 1 mM DTT). After 10 min incubation on ice followed by sonication and centrifugation, cell extracts were precleared by incubation for 30 min at 4°C with 20 μl protein G sepharose - 50% slurry (Amersham). After centrifugation, mix was incubated with 1 μg of anti-SXR or anti-RXR (Santa Cruz Biotechnology) for overnight at 4°C. Immune complexes were collected by incubation with 20 μl protein G sepharose for 1 h and were washed five times with lysis buffer. CPT-11, SN-38 or rifampicin were added during immunoprecipitation and washing steps.

### Chromatin Immunoprecipitation

Cells were rinsed in PBS, cross-linked by adding formaldehyde (1% in PBS) for 10 min and the reaction was stopped with 125 mM glycine for 5 min. After two rinses, cells were scraped in lysis buffer (1% SDS, 10 mM EDTA, 50 mM Tris-HCl pH 8.1) and lysate was subjected to sonication for four 15-second pulses and centrifuged at 14000 g for 15 min at 4°C. The supernatant was diluted 2-fold in buffer (150 mM NaCl, 50 mM Hepes pH 7.9, 1% triton-X100, 0.1% SDS, 2 mM EDTA) and precleared by adding 2 μg of salmon sperm DNA and 20 μl Protein G sepharose, and rotating for 1 h at 4°C. After a brief centrifugation, the supernatant was incubated with 1 μg anti-SXR, anti-RNA polymerase II or anti-phospho-RNA polymerase II (Abcam) overnight at 4°C. The following day, the immune complexes were collected by adding 20 μl Protein G sepharose and incubating for 1 h at 4°C, followed by a centrifugation. The pellet was sequentially washed with low-salt- (150 mM NaCl, 50 mM Hepes pH 7.9, 0.1% SDS, 1% triton X-100, 2 mM EDTA), high-salt- (500 mM NaCl, 50 mM Hepes pH 7.9, 0.1% SDS, 1% triton X-100, 2 mM EDTA), and LiCl- (20 mM tris pH 8.1, 1% NP-40, 1% deoxycholate, 250 mM LiCl, 1 mM EDTA) wash buffers and rinsed with TE buffer. The immune complexes were eluted by 20 min incubation with 1% SDS and 0.5 M NaHCO3. The formaldehyde-induced cross-links were reversed by adding NaCl to a final concentration of 5 mM and incubating at 65°C for 4 h. DNA was then purified using standard phenol/chloroform method. Classical or real-time PCR were carried out with primers amplifying control promoter region or SXR binding sites at -169 bp and -7734 bp from the transcriptional start site of CYP3A4 gene. Data obtained from immunoprecipitated samples were compared with input for classical PCR and normalized to values obtained with the housekeeping gene GAPDH for qPCR.

### RNA extraction and RT-PCR

After 24 h treatment, total RNA was isolated from cells by the acid guanidinium isothiocyanate-phenol-chloroform extraction method [52]. For reverse transcription, 1.3 μg of random hexamers (Amersham) were added to 2 μg of total RNA in a total volume of 15 μl. The mixture was incubated for 5 min at 70°C and then chilled on ice. 4 U/μl of MMLV, 4 mM dNTPs, 1X MMLV buffer and 0.8 U/μl of RNasin (Invitrogen) were added and the whole mixture (50 μl) was further incubated at 37°C for 1 h. Real-time PCR was carried out as describe above. PCR was realized with 5 μl of 1/20 cDNA dilution in a final volume of 10 μl. Total RNA which has not been reverse-transcripted was amplified and used as DNA-free RNA sample control.

### Semi-quantitative PCR

Real-time PCR was carried out using the LightCycler System (Roche Applied Science, Mannheim, Germany). PCR was set up at 4 mM MgCl2, 5 μM of each primer (MWG Biotech, Huntsville, AL, USA), 5 μl of recover DNA and 1X of Master Mix (Roche) in a final volume of 10 μl. Data analysis was essentially performed using "Fit Point Method" in the LightCycler software version 3.5.3. Relative quantification was performed using the comparative Cycle Threshold (CT) method by Fink et al [53]. DNA enrichment quantification or mRNA amplification factor were calculated relative to the presence of GAPDH gene, according to the following equation: EF = 2^-ΔΔCT ^where ΔΔCT = [CT_promoter region _- CT_GAPDH_] _drug _- [CT_promoter region _- CT_GAPDH_] _no treatment _for ChIP experiment, and where ΔΔCT = [CT_target gene_- CT_GAPDH_] _drug _- [CT_target gene_- CT_GAPDH_] _no treatment _for RT-PCR experiment.

The factor was determined from the average of 3 experiments. The sequences of PCR primers are available upon request.

### SXR transcription inhibition

siRNA experiments were carried out with Dharmafect reagent (Thermo Scientific Dharmacon, Lafayette, CO, USA) in LS180 cells, according to the manufacturer's protocol. Cells were transfected 48 h prior treatment with 25 nM siRNA against SXR (SMARTpool Dharmacon) and corresponding amount of siRNA control. SMARTpool is a mixture of four individual siRNA.

HepG2 cells were transfected with 5 nM siRNA from QIAgen (Hilden, Germany) using HiPerfect reagent following manufacturer's instruction (Qiagen). Transfection were performed 48 h and 24 h before treatment.

Target protein expression was controlled by western blot 48 h after siRNA transfection, prior the 24 h drug treatment.

### SRB assay and statistical analysis

The major SXR transcript variant (SXR.1, [54]) cDNA was isolated from human liver cDNA library (Takara Bio Inc., Shiga, Japan) and cloned into the pcDNA3.1 vector (Invitrogen). Cells were transfected with pcDNA3.1-SXR or pcDNA 3.1-control 24 h before treatment. Then, different dilutions of CPT-11 were added to cells and cell survival was measured after 72 h with the sulforhodamine B assay [55]. Each point represents the mean of 3 replicates and the experiment was repeated 3 times. All results are expressed as the mean ± SE and data were analyzed for statistical differences by Student's t test. A p-value of less than 0.05 was considered statistically significant.

## List of abbreviations

The abbreviations used are: SXR: steroid and xenobiotic receptor; CYP: cytochrome p450; RXR: retinoid X receptor; UGT: UDP-glucuronosyltransferase; MDR: multidrug resistance; CPT-11: irinotecan; SN-38: 7-ethyl-10-hydroxycamptothecin; CE: carboxylesterase; BCRP: breast cancer resistance protein; MRP: multidrug resistance protein; CAR: constitutive androstane receptor; FXR: farnesoid X receptor; VDR: Vitamin D Receptor.

## Competing interests

The authors declare that they have no competing interests.

## Authors' contributions

AB carried out the experiments and drafted the manuscript. LP carried out some ChIP assay, some siRNA assay and some survival assay. SCT carried out the WB for siRNA and transfection assays. MBC and EG participated in the conception and design of the study. OC participated in the conception of the study, its design and helped to draft the manuscript. AM conceived of the study, participated in its design and coordination, and helped to draft the manuscript. All authors read and approved the final manuscript.
